# Molecular analysis of genome segment-3 of bluetongue virus serotype 12 isolates from Haryana

**DOI:** 10.14202/vetworld.2017.1389-1393

**Published:** 2017-11-27

**Authors:** Anita Dalal, Sushila Maan, Nitish Bansal, Vinay Kumar, Aman Kumar, Narender Singh Maan, Naresh Kumar Kakker

**Affiliations:** 1 Department of Veterinary Microbiology, College of Veterinary Sciences, Lala Lajpat Rai University of Veterinary and Animal Sciences, Hisar - 125 004, Haryana, India; 2 Department of Animal Biotechnology, College of Veterinary Sciences, Lala Lajpat Rai University of Veterinary and Animal Sciences, Hisar - 125 004, Haryana, India; 3 Department of Animal Nutrition, College of Veterinary Sciences, Lala Lajpat Rai University of Veterinary and Animal Sciences, Hisar - 125 004, Haryana, India; 4 Department of Animal Biotechnology, College of Veterinary Sciences, Lala Lajpat Rai University of Veterinary and Animal Sciences, Hisar - 125 004, Haryana, India

**Keywords:** bluetongue, bluetongue virus-12, genome segment-3, Haryana, real time, serotype, sequencing

## Abstract

**Aim:**

The present study was designed to characterize the genome segment 3 (Seg-3) of bluetongue virus (BTV) serotype 12 isolates from different outbreaks of Bluetongue disease in Haryana, India.

**Materials and Methods:**

Blood and swab samples were collected from goat and sheep suspected to be suffering of BT from different outbreaks from Gurugram, Sirsa, Hisar, and Karnal districts of Haryana. The samples were grown in insect and mammalian cell lines. After preliminary identification, serotyping was done using BTV type-specific quantitative reverse transcription-polymerase chain reaction (qRT-PCR) assays. Sequencing was performed using terminal and walking internal primers specific for Seg-3 on ABI Capillary Sequencer 3130 using a “BigDye cycle sequencing kit.” The obtained sequence data were analyzed with various bioinformatic tools.

**Results:**

Real-time PCR results confirmed the samples to be positive for BTV-12. The Seg-3 of Indian isolates was most closely related to that of a south Indian isolate of BTV-12 from Andhra Pradesh (KC662614) with 97% nucleotide identity.

**Conclusions:**

The study confirmed the circulation of BTV-12 in Haryana, India. The variations shown in genome Seg-3 of BTV-12 isolates may have some significance and need to be further explored.

## Introduction

Bluetongue (BT) is an infectious but non-contagious, economically significant arboviral disease of wild and domestic ruminants, particularly sheep and goats. The etiological agent of the disease is the segmented dsRNA virus having 27 known serotypes and belongs to *Orbivirus* genus of the Reoviridae family [1-4]. In addition, two potential novel BTV serotypes have also been described: One detected in a sheep pox vaccine preparation in Israel [5] and another from Alpaca in South Africa [6]. The virus is not only transmitted by biting midges belonging to different species of *Culicoides* but also horizontally and vertically through oral and semen, respectively [7-9].

BT has been endemic in India with higher incidences in southern states of Peninsular India. Different serotypes and exotic BTV strains have been found circulating in host animal population in this region [10-18]. Therefore, the region may be one of the BTV source areas paving the way to spread across the nation through travel of infected host animals under incubation, insect spp., or reservoir hosts, i.e., cattle and goats [18].

Historically, Haryana state has been the point of attention with respect to BT disease. Two BTV serotypes BTV-1 and BTV-4 have been isolated [19,20]. However, there have been serological evidence of BTV-2, -8, -12, and -16 from Haryana [21]. Recently, BTV-12 and PPRV have been detected as coinfections in sheep and goats in Haryana [22]. The present paper demonstrates the presence of BTV-12 serotype and spread of BTV in small ruminants of four districts of Haryana in 2016 by quantitative reverse-transcription polymerase chain reaction (qRT-PCR) and phylogenetic analysis.

## Materials and Methods

### Ethical approval

Ethical approval was not required as no part of research had been carried out in live laboratory or domestic animals.

### Samples

Four BTV positive samples were used in the present study. Blood and swab samples were collected from goat and sheep suspected to be suffering of BT from different outbreaks from Gurugram, Sirsa, Hisar, and Karnal districts of Haryana (Table-1). The swab samples were resuspended in phosphate-buffered saline (PBS) (pH 7.2). The samples were kept at 4°C till RNA viral genome extracted or further processed for virus isolation in cell culture.

**Table-1:** Details of four BTVpositive samples used in the present study.

Type of animal (sample identity)	Area	Type of sample	Ct value*
Goat (IND2016/118)	Gurugram	Blood	27
Sheep (IND2016/128)	Sirsa	Nasal swab	28
Sheep (IND2016/317)	Hisar	Nasal swab	27.3
Sheep (IND2016/396)	Karnal	Nasal swab	27

* Ct value indicates that the samples were positive for BTV before taking for passage in cell culture. BTV: Bluetongue virus

### Isolation of BTV

RBCs from the heparinized blood collected from BT suspected sheep were washed with PBS and lysed in sterile distilled water. The cell lysate was pelleted, resuspended in PBS, filtered through 0.22 µ millipore filter, inoculated to insect cell line KC cells (*Culicoides sonorensis*), and incubated at 26°C for 10 days. The supernatant along with infected KC cells was further inoculated in BHK-21 cells and incubated at 37°C in the presence of 5% CO_2_. The virus samples were harvested after the appearance of 75-80% cytopathogenic effect visible under an inverted microscope. The swab samples after resuspending in PBS were processed in the same way.

### Viral RNA isolation

Total RNA was extracted from each sample either using a QIAamp Viral RNA Mini Kit (Qiagen, Hilden, Germany) according to the manufacturer’s instructions or using TRIzol Reagent (Life Technologies Inc.) [23]. Similarly, RNA from uninfected tissue culture supernatants and uninfected sheep and goat blood was also extracted for use as negative controls.

### Real-time RT-PCR and RT-PCR

Preliminary identification for BTV-positive samples was done with qRT-PCR using the published protocols targeting Seg-1 [24], Seg-9 [25], and Seg-10 [26]. For determination of BTV serotype, a panel of qRT-PCR assays targeting Seg-2 was used. In addition, the samples were tested for the presence of BTV RNA using conventional gel-based RT-PCR using the serotype-specific Seg-2-based primers [1,27].

### Amplification of genome segment 3 (Seg-3)

The genome Seg-3 of all the isolates of BTV-12 was amplified by conventional PCR using Seg-specific terminal primers (BTV/S3F - GTTAAATTTCCGTAGCCATG and BTV/S3R - GTAAGTGTATTTCCGCTGCTG) using Superscript III one-step RT-PCR system with Platinum Taq high fidelity DNA polymerase kit (Cat. No.: 12574-035). The composition of reaction mixture used as master mix for all PCR reactions was constituted by 12.50 µl one-step RT-PCR master mix, forward and reverse primers 1 µl each, one-step RT enzyme 1.5 µl, and nuclease-free water to make 24 µl. To this reaction, denatured 1 µl of dsRNA template (10 ng/µl) was added and run at thermal protocol for 55°C for 30 min, 94°C for 2 min, 94°C for 15 s, 94°C for 2 min, 50°C for 30-45 s, 68°C for 2-4 min, 68°C for 5 min, and 4°C for infinite.

The PCR amplified products of Seg-3 were run in 1% agarose gel, and the gel bands were excised and purified using Qiagen gel extraction kit according to manufacturer’s protocol. Sequencing of these purified amplicons using terminal and walking internal primers that are specific for genome Seg-3 was done on ABI capillary sequencer 3130 using a “BigDye cycle sequencing kit” in the Department of Animal Biotechnology, LUVAS, Hisar, Haryana.

Contigs were made from the raw data obtained from automated DNA sequencer using software BioEdit and Lasergene 5. The contigs were analyzed using NCBI BLASTn online software tool available on the internet (Website: http://www.ncbi.nlm.nih.gov). ClustalW implemented in BioEdit program was used for multiple sequence alignment, and MEGA-6 program was used to draw phylogenetic tree using Neighbor joining algorithm.

## Results and Discussion

BTV-positive samples with IND2016/118, IND2016/128, IND2016/317, and IND2016/396 sample identity were used for Seg-3 analysis which were collected from four different districts, namely, Gurugram, Sirsa, Hisar, and Karnal of Haryana, respectively (Table-1).

Identification and serotyping were performed by qRT-PCR assays using primers directed against Seg-2 of Indian panel of BTV serotypes (BTV-1, -2, -3, -4, -5, -9, -10, -12, -16, -21, -23, and -24). The results demonstrated low Ct value for BTV-12 serotype ranging from 10-20 after virus isolation in cell culture.

The conventional RT-PCR performed using four overlapping primer pairs of Seg-2 BTV-12 yielded required size products A to D confirming the presence of BTV-12.

## Full-length sequence analyses of genome Seg-3

**Figure-1:**
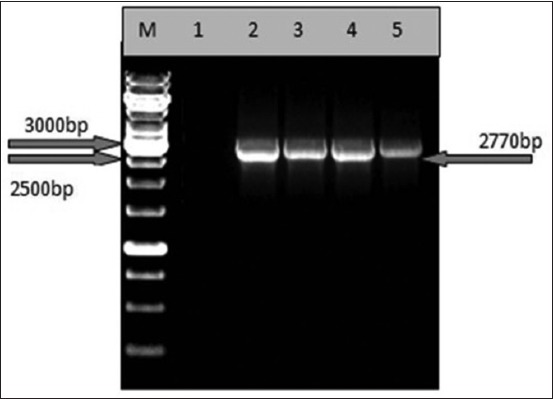
Agarose gel (1%) showing polymerase chain reaction amplified and purified products of segment-3 of all four bluetongue virus isolates used in the present study. Lane M: 1 kb DNA ladder (Thermoscientific); Lane 1: Blank; Lane 2: IND2016/118; Lane 3: IND2016/128; Lane 4: IND2016/317; and Lane 5: IND2016/396.

The genome Seg-3 of the BTV-12 isolates, were PCR amplified and purified. The gel purified eluted products were again confirmed in 1% agarose for purity and size (Figure-1). The sequences obtained for Seg-3 from these purified products were analyzed using bioinformatics tools described in the material and methods section. The consensus sequences for Seg-3 were generated for Seg-3 for all the five isolates.

**Figure-2:**
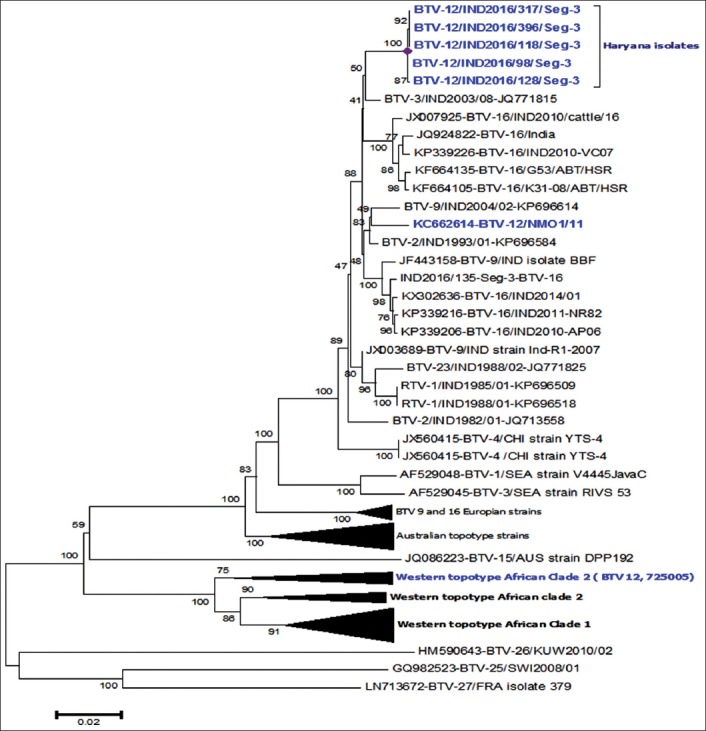
Unrooted neighbor joining tree for segment-3 of bluetongue virus (BTV) isolates. It shows relationships of BTV isolates from Haryana with those of other isolates from worldwide. The tree is generated with MEGA-6 software using default parameters.

Seg-3 of BTV is 2772 bp long genome encoding the highly conserved 901 a subcore protein VP3 of virus particle (regardless of serotype or topotype). In the present study, all the BTV isolates gave 2769 bp long sequence of VP3/Seg-3. Further, a significant observation has been made in the 3’ end of the Seg-3, i.e., there is marked insertional mutation “thymine” nucleotide at 2765 position. Transition has also been noted at 2753 and 2764 with guanine to adenine and cytosine to thymine, respectively. The Seg-3 sequence of all the four BTV-12 isolates, i.e., IND2016/118, IND2016/128, IND2016/317, and IND2016/396 was most closely related to a South Indian isolate INDAPADBNMO1/11(KC662614.1) of BTV-12 from Andhra Pradesh with 97% nucleotide identity (Figure-2). On comparison with western topotype BTV-12 isolate 75005 (JX272501.1) from South Africa, the present BTV isolates demonstrated 80.6-80.7% identity. Scientists reported that Seg-3 of BTV-1 exchanged with Seg-3 of BTV-26 serotype by reverse genetics, resulted in restricted replication in KC cells, thereby indicating the role of Seg-3 in vector transmission of the virus [28]. Therefore, further studies are required to evaluate the functional role of structural protein VP3 in BTV life cycle in addition to forming subcore of the virion.

In India, the very first isolation and second complete genome sequence of BTV-12, INDAPADBNMO1/11 (KC662612-KC662621) were from Andhra Pradesh [17], after a Taiwan BTV-12 whole genome sequence [29]. The serological presence of BTV-12 by detection of anti-BTV-12 antibodies in sheep and cattle form Andhra Pradesh, Gujarat, and Haryana has earlier been reported [21]. Moreover, there have been no reports about the existence of eastern topotype for this serotype. The Asian and African isolates have been found to be closely related, and it has been speculated that these isolates of BTV-12 have a recent common ancestry. Moreover, there has been a correlation with modified live vaccines used in the South Africa and USA against BTV-12 [17,18].

In conclusion, less identity and marked variations in genome Seg-3 of BTV isolates of Haryana origin from previously published sequences of BTV-12 from southern India may have some significance that needs to be further explored.

## Authors’ Contributions

AD, NKK, and SM: Analyzed the data and drafted the manuscript. AD, SM, NKK, NSM, VK, and NB: Contacted the officials of State AH department and collection of samples. AD, VK, AK, and NB: Conducted the experiments. SM, NSM, and AK: Provided reagents and materials. AD, SM, NKK, and NSM: Proof read the manuscript and provided the guidance. All authors read and approved the final manuscript.

## References

[1] Hofmann M.A, Renzullo S, Mader M, Chaignat V, Worwa G, Thuer B (2008). Genetic characterization of toggenburg *Orbivirus* a new bluetongue virus, from goats, Switzerland. Emerg. Infect. Dis.

[2] Maan S, Maan N.S, Nomikou K, Batten C, Antony F, Belaganahalli M.N, Mertens P.P.C (2011). Novel bluetongue virus serotype from Kuwait. Emerg. Infect. Dis.

[3] Sun E.C, Huang L.P, Xu Q.Y, Wang H.X, Xue X.M, Lu P, Li W.J, Liu W, Bu Z.G, Wu D.L (2016). Emergence of a novel bluetongue virus serotype, China 2014. Transbound. Emerg. Dis.

[4] Zientara S, Sailleau C, Viarouge C, Höper D, Beer M, Jenckel M, Hoffmann B, Romey A, Kassimi L.B, Fablet A, Vitour D, Bréard E (2014). Novel bluetongue virus in goats, Corsica, France, 2014. Emerg. Infect. Dis.

[5] Bumbarov V, Golender N, Erster O, Khinich Y (2016). Detection and isolation of bluetongue virus from commercial vaccine batches. Vaccine.

[6] Wright I.M (2014). Serological and Genetic Characterisation of Putative New Serotypes of Bluetongue Virus and Epizootic Haemorrhagic Disease Virus Isolated from an Alpaca.

[7] Rasmussen L.D, Savini G, Lorusso A, Bellacicco A, Palmarini M, Caporale M, Rasmussen T.B, Belsham G.J, Bøtner A (2013). Transplacental transmission of field and rescued strains of BTV-2 and BTV-8 in experimentally infected sheep. Vet. Res.

[8] Savini G, Lorusso A, Paladini C, Migliaccio P, Di Gennaro A, Di Provvido A, Scacchia M, Monaco F (2014). Bluetongue serotype 2 and 9 modified live vaccine viruses as causative agents of abortion in livestock:A retrospective analysis in Italy. Transbound. Emerg. Dis.

[9] van der Sluijs M.T, de Smit A.J, Moormann R.J (2016). Vector independent transmission of the vector-borne bluetongue virus. Crit. Rev. Microbiol.

[10] Gollapalli S.R, Mallavarapu S, Uma M, Rao P.P, Susmitha B, Prasad P.U, Chaitanya P, Prasad G, Hegde N.R, Reddy Y.N (2012). Sequences of genes encoding type-specific and group-specific antigens of an Indian isolate of bluetongue virus serotype 10 (BTV-10) and implications for their origin. Transbound. Emerg. Dis.

[11] Maan N.S, Maan S, Guimera M, Nomikou K, Morecroft E, Pullinger G, Belaganahalli M.N, Mertens P.P.C (2012). The genome sequence of a reassortant bluetongue virus serotype 3 from India. J. Virol.

[12] Maan N.S, Maan S, Guimera M, Pullinger G, Singh K.P, Nomikou K, Belaganahalli M.N, Mertens P.P.C (2012). Complete genome sequence of an isolate of bluetongue virus serotype 2, demonstrating circulation of a Western topotype in southern India. J. Virol.

[13] Maan N.S, Maan S, Nomikou K, Guimera M, Pullinger G, Singh K.P, Belaganahalli M.N, Mertens P.P.C (2012). The genome sequence of bluetongue virus Type 2 from India:Evidence for reassortment between eastern and western topotype field strains. J. Virol.

[14] Maan N.S, Maan S, Nomikou K, Prasad G, Singh K.P, Belaganahalli M.N, Mertens P.P.C (2012). Full genome sequence of bluetongue virus serotype 1 from India. J. Virol.

[15] Maan S, Maan N.S, Guimera M, Nomikou K, Singh K.P, Pullinger G, Belaganahalli M.N, Mertens P.P.C (2012f). Genome sequence of a reassortant strain of bluetongue virus serotype 23 from western India. J. Virol.

[16] Rao P.P, Reddy Y.V, Meena K, Karunasree N, Susmitha B, Uma M, Prasad P.U.V, Chaitanya P, Reddy Y.N, Hegde N.R (2012). Genetic characterization of bluetongue virus serotype 9 isolates from India. Virus Genes.

[17] Rao P, Reddy Y, Hegde N (2015). Isolation and complete genome sequencing of bluetongue virus serotype 12 from India. Transbound. Emerg. Dis.

[18] Rao P.P, Hegde N.R, Reddy Y.N, Krishnajyothi Y, Reddy Y.V, Susmitha B, Gollapalli S.R, Putty K, Reddy G.H (2016). Epidemiology of bluetongue in India. Transbound. Emerg. Dis.

[19] Jain N.C, Sharma R, Prasad G (1986). Isolation of bluetongue virus from sheep in India. Vet. Rec.

[20] Uppal P, Vasudevan B (1980). Occurrence of bluetongue in India. Ind. J. Comp. Microbiol. Immunol. Infect. Dis.

[21] Prasad G, Sreenivasulu D, Singh K.P, Metens P.P.C, Maan S, Mertens P.P.C, Baylis M, Mellor P.S (2009). Bluetongue in the Indian subcontinent. Bluetongue.

[22] Maan S, Kumar A, Gupta A.K, Dalal A, Chaudhary D, Gupta T.K, Bansal N, Kumar V, Batra K, Sindhu N, Kumar A, Mahajan N.K, Maan N.S, Mertens P.P.C (2017). Concurrent infection of bluetongue and peste-despetits-ruminants virus in small ruminants in Haryana State of India. Transbound. Emerg. Dis.

[23] Attoui H, Billoir F, Cantaloube J.F, Biagini P, de Micco P, de Lamballerie X (2000). Strategies for the sequence determination of viral dsRNA genomes. J. Virol. Methods.

[24] Shaw A.E, Monaghan P, Alpar H.O, Anthony S, Darpel K.E, Batten C.A, Guercio A, Alimena G, Vitale M, Bankowska K, Carpenter S, Jones H, Oura C.A, King D.P, Elliott H, Mellor P.S, Mertens P.P.C (2007). Development and initial evaluation of a real-time RT-PCR assay to detect bluetongue virus genome segment 1. J. Virol. Methods.

[25] Maan S, Maan N.S, Belaganahalli M.N, Potgieter A.C, Kumar V, Batra K, Wright I.M, Kirkland P.D, Mertens P.P.C (2016). Development and evaluation of real time RT-PCR assays for detection and typing of bluetongue virus. PLoS One.

[26] Orru G, Ferrando M.L, Meloni M, Liciardi M, Savini G, De Santis P (2006). Rapid detection and quantitation of bluetongue virus (BTV) using a molecular beacon fluorescent probe assay. J. Virol. Methods.

[27] Maan N.S, Maan S, Belaganahalli M.N, Ostlund E.N, Johnson D.J, Nomikou K, Mertens P.P.C (2012). Identification and differentiation of the twenty six bluetongue virus serotypes by RT-PCR amplification of the serotype-specific genome segment 2. PLoS One.

[28] Pullinger G.D, Guimerà B.M, Nomikou K, Boyce M, Attoui H, Mertens P.P.C (2016). Identification of the genome segments of bluetongue virus serotype 26 (isolate KUW2010/02) that restrict replication in a *Culicoides sonorensis* cell line (KC cells). PLoS One.

[29] Lee F, Ting L.J, Lee M.S, Chang W.M, Wang F.I (2011). Genetic analysis of two taiwanese bluetongue viruses. Vet. Microbiol.

